# Diagnostic Utility of High-Performance Liquid Chromatography and Its Correlation With Hematological Indices in the Evaluation of Hemoglobinopathies

**DOI:** 10.7759/cureus.109181

**Published:** 2026-05-19

**Authors:** Radha Sharma, Nilofer Khayyam, Shiv prakash Sharma, Yogesh K Gupta, Vivek Sharma

**Affiliations:** 1 Pathology, College of Medical Sciences (CMS), Rajasthan University of Health Sciences (RUHS), Jaipur, IND; 2 Microbiology, College of Medical Sciences (CMS), Rajasthan University of Health Sciences (RUHS), Jaipur, IND; 3 Preventive Medicine, College of Medical Sciences (CMS), Rajasthan University of Health Sciences (RUHS), Jaipur, IND; 4 Pathology and Laboratory Medicine, College of Medical Sciences (CMS), Rajasthan University of Health Sciences (RUHS), Jaipur, IND

**Keywords:** beta thalassemia trait, hba2, hematological indices, hemoglobinopathies, : high-performance liquid chromatography, microcytic anemia

## Abstract

Introduction: Hemoglobinopathies are common genetic disorders in India and frequently present as microcytic hypochromic anemia, often mimicking iron deficiency anemia. High-performance liquid chromatography (HPLC) enables accurate quantification of hemoglobin fractions and facilitates reliable diagnosis.

Methodology: This cross-sectional study included 150 patients with microcytic hypochromic anemia (mean corpuscular volume (MCV) < 80 fL, mean corpuscular hemoglobin (MCH) < 27 pg) and clinical suspicion of hemoglobinopathy. Complete blood count and HPLC (Bio-Rad D10, Bio-Rad Laboratories, Hercules, CA) were performed. Hemoglobin fractions were quantified, and hematological indices were correlated with HPLC findings. Statistical analysis was performed using independent t-tests and chi-square tests.

Results: Abnormal hemoglobin patterns were identified in 100 (66.7%) patients. Beta thalassemia trait was the most common finding, seen in 63 patients (42.0%), and was characterized by elevated HbA2 levels (mean: 5.2% ± 0.8%). These patients showed significantly lower MCV and MCH values and higher red blood cell (RBC) counts compared with those with normal HPLC patterns (*P* < 0.001). Beta-thalassemia major was identified in 12 (8.0%) with markedly elevated fetal hemoglobin (HbF) (mean: 85.6% ± 8.2%). Structural variants, including HbS trait (7, 4.7%) and homozygous HbE (6, 4.0%), were also detected based on characteristic HPLC patterns.

Conclusions: HPLC is a reliable and precise diagnostic tool for hemoglobinopathies, with a strong correlation to hematological indices. Its use enables accurate differentiation of thalassemia carriers from other causes of microcytic anemia, supporting its role in routine diagnostic evaluation.

## Introduction

Hemoglobinopathies constitute a major public health burden in India, with an estimated 30-40 million carriers of beta-thalassemia and a significant prevalence of structural hemoglobin variants such as HbS, HbE, and HbD [1]. These disorders commonly present as microcytic hypochromic anemia, a hematological picture that closely resembles iron deficiency anemia and often leads to diagnostic uncertainty [2]. Accurate identification is essential for appropriate clinical management, genetic counseling, and prevention of severe disease through prenatal screening programs [3].

Conventional diagnostic methods, including hemoglobin electrophoresis, have limitations such as poor resolution and inability to precisely quantify hemoglobin fractions, particularly HbA2 and HbF [4]. High-performance liquid chromatography (HPLC) has emerged as a superior alternative, offering automated analysis, high resolution, and accurate quantification of hemoglobin fractions along with presumptive identification of variants based on retention times [5].

Measurement of HbA2 plays a pivotal role in the diagnosis of beta-thalassemia trait, with levels of 3.5%-3.9% or higher considered diagnostic [6]. However, optimal interpretation of HPLC findings requires correlation with hematological indices, as beta-thalassemia trait is typically characterized by microcytosis, hypochromia, and a relatively elevated red blood cell (RBC) count [7]. In regions like India, where both hemoglobinopathies and nutritional anemias are highly prevalent, this combined approach is crucial for accurate differentiation and prevention of misdiagnosis [8].

The present study aimed to evaluate the diagnostic utility of HPLC in detecting hemoglobinopathies and assess its correlation with hematological indices in patients presenting with microcytic hypochromic anemia.

## Materials and methods

Study design and setting

This cross-sectional, hospital-based observational study was conducted in the Department of Pathology at RUHS College of Medical Sciences and associated hospitals, Jaipur, India, from August 2024 to February 2025. Ethical approval was obtained from the Institutional Ethics Committee (approval number: RUHS-CMS/Ethics Comm./2024/338).

Study population

A total of 150 patients with microcytic hypochromic anemia and clinical suspicion of hemoglobinopathy were included. The sample size was calculated using the formula *n* = 4*pq*/*d*², assuming a prevalence of 10% (*p* = 0.10, *q* = 0.90, *d* = 0.05), yielding a minimum sample size of 144, which was rounded to 150.

Inclusion and exclusion criteria

Patients of all age groups presenting with microcytic hypochromic anemia (mean corpuscular volume (MCV) <80 fL and mean corpuscular hemoglobin (MCH) < 27 pg) with clinical suspicion of hemoglobinopathy based on family history, ethnic background, or characteristic clinical features were included. Children and parents of known cases were also considered. Patients who had received blood transfusions within the preceding three months or those unwilling to provide informed consent were excluded.

Sample collection and processing

Five milliliters of venous blood were collected under aseptic precautions in ethylenediaminetetraacetic acid (EDTA) anticoagulant tubes and processed within two hours of collection.

Complete blood count analysis

Complete blood count was performed using an automated hematology analyzer (Sysmex XT-4000i, Kobe, Japan). Parameters recorded included hemoglobin, RBC count, hematocrit, mean corpuscular volume, mean corpuscular hemoglobin, mean corpuscular hemoglobin concentration (MCHC), and red cell distribution width.

High-performance liquid chromatography

Hemoglobin fraction analysis was carried out using the Bio-Rad D10 HPLC system (Bio-Rad Laboratories, Hercules, CA), based on cation-exchange chromatography with gradient elution. Calibration and quality control were performed as per manufacturer recommendations. Hemoglobin fractions, including HbA, HbA2, HbF, and abnormal variants, were quantified based on retention times and peak areas.

Diagnostic criteria

Beta-thalassemia trait was diagnosed when HbA2 exceeded 3.9% in the presence of microcytic hypochromic indices. Beta-thalassemia major was defined by markedly elevated HbF (>70%) with reduced or absent HbA. The HbS trait was identified by a characteristic peak in the S-window comprising approximately 35%-40% of total hemoglobin. HbE homozygous state was diagnosed by a predominant HbE peak (>80%) with associated HbF elevation. Hereditary persistence of fetal hemoglobin was considered when HbF ranged between 15% and 30% without significant elevation of HbA2.

Statistical analysis

Data were analyzed using SPSS version 22.0 (IBM Corp., Armonk, NY). Continuous variables were expressed as mean ± standard deviation, and categorical variables were presented as frequencies and percentages. Independent t-tests were used to compare continuous variables between groups, while chi-square tests were used to compare categorical variables. A *P*-value of less than 0.05 was considered statistically significant.

## Results

Spectrum of hemoglobinopathies

A total of 150 patients with microcytic hypochromic anemia were evaluated, with a mean age of 26.4 ± 15.8 years. Females comprised 82 (54.7%) and males 68 (45.3%) of the study population. Abnormal hemoglobin patterns on HPLC were identified in 100 (66.7%) patients, while 50 (33.3%) showed normal patterns. Beta-thalassemia trait was the most common abnormality, detected in 63 (42.0%) patients, followed by beta-thalassemia major in 12 (8.0%) patients. Structural hemoglobin variants included HbS trait in 7 (4.7%) and HbE homozygous cases in 6 (4.0%) patients. Hereditary persistence of fetal hemoglobin was identified in 4 (2.7%) patients, and other rare variants were observed in 8 (5.3%) patients.

Hematological indices in beta-thalassemia trait

Patients with beta-thalassemia trait demonstrated consistent microcytosis and hypochromia, with a majority showing relatively elevated RBC counts. The mean hemoglobin level was 9.8 ± 1.4 g/dL. The distribution of hematological parameters in beta-thalassemia trait cases is summarized in Table 1.

**Table 1:** Descriptive distribution of hematological parameters in cases with beta-thalassemia trait (n = 63). Hb, hemoglobin; MCV, mean corpuscular volume; MCH, mean corpuscular hemoglobin; MCHC, mean corpuscular hemoglobin concentration; RBC, red blood cell

Parameter	Range	Number of cases (*n*)	Percentage (%)
Hemoglobin	<9 g/dL	22	34.9
	9-10.9 g/dL	28	44.4
	≥11 g/dL	13	20.6
MCV	<80 fL	63	100.0
MCH	<27 pg	63	100.0
MCHC	>32%	55	87.3
RBC count	>4.8 million/μL	42	66.6

Hemoglobin fractions in different hemoglobinopathies

All 63 (100%) beta-thalassemia trait cases showed elevated HbA2 levels (>3.9%), with a mean of 5.2 ± 0.8% compared with 2.7% ± 0.4% in patients with normal HPLC patterns, a statistically significant difference (*t* = 18.92, *P* < 0.001). Beta-thalassemia major cases demonstrated markedly elevated HbF (mean 85.6% ± 8.2%) with reduced HbA levels. HbE homozygous cases showed predominant HbE fractions (mean 82.3% ± 6.1%) with mild HbF elevation, while HbS trait cases demonstrated characteristic HbS fractions (mean 38.5% ± 3.2%). Hereditary persistence of fetal hemoglobin cases showed moderately elevated HbF without significant HbA2 elevation. Hemoglobin fraction profiles across different hemoglobinopathies are summarized in Table 2.

**Table 2:** Hemoglobin fractions in different hemoglobinopathies. *One-way analysis of variance (ANOVA) comparing mean hemoglobin fractions (HbA, HbA2, HbF) across diagnostic groups.

Diagnosis	Number of cases (*n*)	HbA (%), mean ± SD	HbA2 (%), mean ± SD	HbF (%), mean ± SD	Abnormal Hb (%), mean ± SD	Test statistics*
Beta-thalassemia trait	63	91.2 ± 3.5	5.2 ± 0.8	1.8 ± 1.2	-	*F *= 156.72, *P *< 0.001
Beta-thalassemia major	12	5.8 ± 4.2	2.3 ± 0.6	85.6 ± 8.2	-	
HbE homozygous	6	10.5 ± 5.1	3.4 ± 0.7	4.8 ± 2.1	82.3 ± 6.1 (HbE)	
HbS trait	7	56.2 ± 4.8	3.1 ± 0.5	1.5 ± 0.8	38.5 ± 3.2 (HbS)	
HPFH	4	76.4 ± 5.2	2.4 ± 0.4	20.5 ± 4.8	-	

Comparison of hematological indices between groups

Comparison between the beta-thalassemia trait and normal HPLC groups revealed significantly lower MCV (*t* = 12.45, *P* < 0.001) and MCH (*t *= 10.87, *P *< 0.001), along with significantly higher RBC counts (*t* = 8.34, *P *< 0.001) and HbA2 levels (*t *= 18.92, *P *< 0.001) in the beta-thalassemia trait group. Hemoglobin levels and MCHC did not differ significantly between the groups. A comparison of hematological indices between beta-thalassemia trait and normal HPLC groups is presented in Table 3.

**Table 3:** Comparison of hematological indices: beta-thalassemia trait versus normal HPLC. *Statistically significant (*P* < 0.05); an independent t-test was used for analysis. HPLC, high-performance liquid chromatography

Parameter	Beta-thalassemia trait (*n* = 63), mean ± SD	Normal HPLC (*n *= 50), mean ± SD	*t*-value	*P*-value
Hemoglobin (g/dL)	9.8 ± 1.4	10.2 ± 1.6	1.42	0.158
MCV (fL)	64.3 ± 4.2	72.5 ± 5.8	12.45	<0.001*
MCH (pg)	22.1 ± 1.8	25.4 ± 2.1	10.87	<0.001*
MCHC (%)	33.2 ± 1.1	32.8 ± 1.3	1.76	0.081
RBC count (million/μL)	5.2 ± 0.6	4.4 ± 0.5	8.34	<0.001*
HbA2 (%)	5.2 ± 0.8	2.7 ± 0.4	18.92	<0.001*

Diagnostic performance of HPLC

HPLC demonstrated high diagnostic accuracy in identifying hemoglobinopathies. Elevated HbA2 levels in conjunction with microcytic hypochromic indices reliably identified beta-thalassemia trait, while markedly elevated HbF with reduced HbA was diagnostic of beta-thalassemia major. Structural variants were identified based on characteristic retention times and peak patterns.

## Discussion

This study demonstrates that HPLC is a reliable and effective tool for the diagnosis of hemoglobinopathies, with a strong correlation between hemoglobin fraction analysis and hematological indices in patients presenting with microcytic hypochromic anemia.

Beta-thalassemia trait was the most common abnormality, identified in 63 (42.0%) patients, based on elevated HbA2 levels (>3.9%) [6]. These patients consistently exhibited the characteristic hematological profile of microcytosis, hypochromia, and relatively elevated RBC counts. The mean HbA2 level of 5.2% ± 0.8% observed in this study is comparable to previous Indian studies, which have reported similar ranges in affected individuals [9,10]. This highlights the robustness of HbA2 as a diagnostic marker when measured using HPLC.

Differentiating beta-thalassemia trait from iron deficiency anemia is clinically important, as both conditions present with similar red cell indices. In the present study, patients with normal HPLC patterns demonstrated significantly lower HbA2 levels compared to those with beta-thalassemia trait (*P *< 0.001), reinforcing the role of HPLC in preventing misdiagnosis. Previous large-scale analyses have similarly emphasized the importance of HPLC in populations with a high prevalence of both conditions [11].

Beta thalassemia major cases showed markedly elevated HbF levels with reduced HbA, reflecting the underlying defect in beta-globin chain synthesis [12]. This pattern is well documented and supports the utility of HPLC not only in diagnosis but also in clinical characterization of disease severity and monitoring. Structural hemoglobin variants were also reliably identified. HbS trait cases demonstrated characteristic HbS fractions consistent with heterozygous states [13,14], while HbE homozygous cases showed predominant HbE fractions with mild HbF elevation, reflecting their thalassemic phenotype [15].

Hereditary persistence of fetal hemoglobin cases showed elevated HbF without significant anemia or alteration in red cell indices. Although HPLC facilitates detection, differentiation from other conditions such as delta-beta thalassemia requires additional clinical and family-based evaluation [4].

The findings of this study underscore the importance of combining HPLC with hematological indices for accurate diagnosis. In resource-limited settings with a high burden of hemoglobinopathies, this integrated approach can significantly improve diagnostic accuracy and guide appropriate clinical management and genetic counseling.

Limitations

Iron studies were not routinely performed in patients with normal HPLC patterns, limiting confirmation of iron deficiency. Molecular characterization of rare variants and compound heterozygous states was not undertaken. The study population was selected based on clinical suspicion, which may overestimate prevalence compared to community-based data. The modest sample size limited subgroup analysis of less common hemoglobin variants, and family studies were not systematically performed.

## Conclusions

HPLC is a reliable and precise diagnostic tool for hemoglobinopathies, demonstrating a strong correlation with hematological indices. Its ability to accurately quantify hemoglobin fractions enables effective differentiation of thalassemia carriers from other causes of microcytic hypochromic anemia. Incorporating HPLC into routine diagnostic evaluation can significantly improve diagnostic accuracy and support early detection and appropriate clinical management in high-burden settings.

## References

[1] Weatherall D (2011). The inherited disorders of haemoglobin: an increasingly neglected global health burden. Indian J Med Res.

[2] Clarke GM, Higgins TN (2000). Laboratory investigation of hemoglobinopathies and thalassemias: review and update. Clin Chem.

[3] Colah RB, Gorakshakar AC, Nadkarni AH (2011). Invasive and non-invasive approaches for prenatal diagnosis of haemoglobinopathies: experiences from India. Indian J Med Res.

[4] Bain BJ, Daniel Y, Henthorn J (2023). Significant haemoglobinopathies: A guideline for screening and diagnosis: A British Society for Haematology Guideline: A British Society for Haematology Guideline. Br J Haematol.

[5] Gupta PK, Kumar H, Kumar S, Jaiprakash M (2009). Cation exchange high performance liquid chromatography for diagnosis of hemoglobinopathies. Med J Armed Forces India.

[6] Bain BJ (2011). Haemoglobinopathy diagnosis: algorithms, lessons and pitfalls. Blood Rev.

[7] Philip J, Sarkar RS, Kushwaha N (2013). Microcytic hypochromic anemia: should high performance liquid chromatography be used routinely for screening anemic and antenatal patients?. Indian J Pathol Microbiol.

[8] Colah RB, Surve R, Sawant P (2007). HPLC studies in hemoglobinopathies. Indian J Pediatr.

[9] Rao S, Kar R, Gupta SK, Chopra A, Saxena R (2010). Spectrum of haemoglobinopathies diagnosed by cation exchange-HPLC and modulating effects of nutritional deficiency anaemias from north India. Indian J Med Res.

[10] Khera R, Singh T, Khuana N, Gupta N, Dubey AP (2015). HPLC in characterization of hemoglobin profile in thalassemia syndromes and hemoglobinopathies: a clinicohematological correlation. Indian J Hematol Blood Transfus.

[11] Sachdev R, Dam AR, Tyagi G (2010). Detection of Hb variants and hemoglobinopathies in Indian population using HPLC: report of 2600 cases. Indian J Pathol Microbiol.

[12] Mondal SK, Mandal S (2016). Prevalence of thalassemia and hemoglobinopathy in eastern India: a 10-year high-performance liquid chromatography study of 119,336 cases. Asian J Transfus Sci.

[13] Vichinsky EP (2005). Changing patterns of thalassemia worldwide. Ann N Y Acad Sci.

[14] Chandrashekar V, Soni M (2011). Hemoglobin disorders in South India. ISRN Hematol.

[15] Cao A, Kan YW (2013). The prevention of thalassemia. Cold Spring Harb Perspect Med.

